# IRT/IRT as a newborn cystic fibrosis screening method: optimal cutoff points for a mixed population

**DOI:** 10.1590/0102-311XEN150623

**Published:** 2024-08-26

**Authors:** Carolina Godoy, Pedro Paulo Brito, Tatiana Amorim, Edna Lúcia Souza, Ney Boa-Sorte

**Affiliations:** 1 Programa de Pós-graduação em Medicina e Saúde, Universidade Federal da Bahia, Salvador, Brasil.; 2 Escola Bahiana de Medicina e Saúde Pública, Salvador, Brasil.; 3 Associação de Pais e Amigos dos Excepcionais, Salvador, Brasil.; 4 Universidade do Estado da Bahia, Salvador, Brasil.

**Keywords:** Cystic Fibrosis, Neonatal Screening, Data Accuracy, Diagnostic Screening Programs, National Health Programs, Fibrose Cística, Triagem Neonatal, Confiabilidade dos Dados, Programas de Triagem Diagnóstica, Programas Nacionais de Saúde, Fibrosis Quística, Tamizaje Neonatal, Exactitud de los Datos, Programas de Detección Diagnóstica, Programas Naionales de Salud

## Abstract

The Brazilian Unified National Health System (SUS) has incorporated newborn screening for cystic fibrosis since 2001. The protocol involves two samples of immunoreactive trypsinogen (IRT1/IRT2). This study aims to analyze fixed and floating values at the first and second IRT (IRT1/IRT2) cutoff points and assess the accuracy of the IRT/IRT methodology in a population from Northeastern Brazil. Descriptive, individual-level data from the newborn screening reference service data system (2013-2017) were used in this observational population study. The sensitivity, specificity, and positive predictive values (PPV) for the protocol were calculated. The best cutoff point was determined using the Youden’s index. The previous year’s cut-off values for the IRT1 and IRT2 99.4-, 99.5-, 99.6-, and 99.7-percentiles were utilized for the floating cutoff. During the studied period, 840,832 newborns underwent screening for cystic fibrosis, obtaining 49 cystic fibrosis diagnoses: 39 by newborn screening (79.6%) and 10 (20.4%) by clinical suspicion (false negative). The sensitivity, specificity, and PPV of the protocol totaled 79.6%, 99.9%, and 6.1%, respectively. No proposed cutoff for IRT1 performed better than the current one. IRT2 performed similarly to the current protocol at a cutoff point of 90ng/mL, showing the appropriate sensitivity and specificity while reducing the frequency of false positives. The protocol to screen newborns for cystic fibrosis had low sensitivity, a predictive positive value, and a high number of false positives and negatives. A floating cut point for IRT1 or IRT2 seems to constitute no viable option. However, changing the IRT2 cut point from 70ng/mL to 90ng/mL seems to have advantages and should undergo consideration.

## Resumo

A triagem neonatal para fibrose cística oi incorporada ao Sistema Único de Saúde (SUS) em 2001. O protocolo envolve duas amostras de tripsinogênio imunorreativo (TIR/TIR). O objetivo foi analisar os valores fixos e flutuantes no primeiro e segundo pontos de corte da TIR (TIR1/TIR2) e avaliar a acurácia da metodologia TIR/TIR em uma população do nordeste brasileiro. Trata-se de um estudo observacional de base populacional que inclui dados descritivos em nível individual obtidos retrospectivamente do Serviço de Referência em Triagem Neonatal (2013-2017). Foram calculados a sensibilidade, a especificidade e o valor preditivo positivo (VPP) do protocolo. O melhor ponto de corte foi determinado pelo índice de Youden. Os pontos de corte do ano anterior para os percentis TIR1 e TIR2 de 99,4, 99,5, 99,6 e 99,7 foram utilizados para o ponto de corte flutuante. No período do estudo, 840.832 recém-nascidos foram submetidos à triagem neonatal para fibrose cística, com 49 diagnósticos de fibrose cística, sendo 39 pela triagem neonatal (79,6%) e 10 (20,4%) por suspeita clínica (falso-negativos). A sensibilidade, a especificidade e o VPP do protocolo de triagem neonatal para fibrose cística foram de 79,6%, 99,9% e 6,1%, respectivamente. Nenhum dos pontos de corte propostos para a TIR1 mostrou-se melhor do que o atual. A TIR2 teve desempenho semelhante ao atual no ponto de corte de 90ng/mL, demonstrando sensibilidade e especificidade adequadas, ao mesmo tempo que reduziu a frequência de falsos positivos. A triagem neonatal para fibrose cística apresentou valores baixos de sensibilidade e VPP, e número elevado de falso-positivos e negativos. Um ponto de corte flutuante para TIR1 ou TIR2 não parece ser uma opção viável. No entanto, a mudança do ponto de corte da TIR2 de 70ng/mL para 90ng/mL parece ter vantagens e deve ser considerada.

## Resumen

El tamizaje neonatal de fibrosis quística fue incorporado al Sistema Único de Salud (SUS) en el 2001. El protocolo implica dos muestras de tripsinógeno inmunorreactivo (TIR/TIR). El objetivo fue analizar los valores fijos y flotantes en el primer y segundo puntos de corte de la TIR (TIR1/TIR2) y evaluar la precisión de la metodología TIR/TIR en una población del Nordeste brasileño. Se trata de un estudio observacional de base poblacional que incluye datos descriptivos a nivel individual obtenidos retrospectivamente del Servicio de Referencia en Tamizaje Neonatal (2013-2017). Se calcularon la sensibilidad, la especificidad y el valor predictivo positivo (VPP) del protocolo. El mejor punto de corte lo determinó el índice de Youden. Para el punto de corte flotante, se utilizaron los puntos de corte del año anterior para los percentiles TIR1 y TIR2 de 99,4, 99,5, 99,6 y 99,7. Durante el período de estudio, 840.832 recién nacidos fueron sometidos a tamizaje neonatal para fibrosis quística, con 49 diagnósticos de fibrosis quística, 39 de los cuales por la tamizaje neonatal (79,6%) y 10 (20,4%) por sospecha clínica (falsos negativos). La sensibilidad, la especificidad y el VPP del protocolo tamizaje neonatal para fibrosis quística fueron del 79,6%, 99,9% y 6,1%, respectivamente. Ninguno de los puntos de corte propuestos para la TIR1 resultó ser mejor que el actual. La TIR2 tuvo un desempeño similar al actual en el punto de corte de 90ng/mL, lo que demuestra sensibilidad y especificidad adecuadas, a la vez que redujo la frecuencia de falsos positivos. El tamizaje neonatal para fibrosis quística presentó valores bajos de sensibilidad y VPP, y un elevado número de falsos positivos y negativos. Un punto de corte flotante para TIR1 o TIR2 no parece ser una opción viable. Sin embargo, cambiar el punto de corte de la TIR2 de 70ng/mL a 90ng/mL parece tener ventajas y debe tenerse en cuenta.

## Introduction

Newborn screening (NBS) provides the opportunity to diagnose diseases with clinical and economic importance to the health system in their early stages (preferably in their asymptomatic states) so early treatment may better benefit patients in the later stages of their diseases ^1^ ^,^ ^2^. An accurate test or screening approach must be developed for a disease to support NBS. Ultimately, it involves balancing a sufficiently high sensitivity and an adequate specificity ^2^ ^,^ ^3^ ^,^ ^4^.

Cystic fibrosis (CF), an inherited autosomal recessive disease with a chronic course and poor prognosis, is characterized by a defective gene that encodes the cystic fibrosis transmembrane conductance regulator (CFTR) protein. This condition unbalances water transport and sodium reabsorption, resulting in dysfunction in multiple organs ^5^ ^,^ ^6^.

The incidence of cystic fibrosis varies greatly around the world. In Europe, it occurs in 1:2,500 live births, whereas the average incidence in North America totals 1:3,500 ^7^. According to the annual report of the Brazilian Group for Cystic Fibrosis Studies, based on the CF-NBS, the incidence of the disease in 2021 totaled 1/19,860 live births ^8^. Many Brazilian states lack such data, especially those with the lowest development indicators, such as in Northern and Northeastern Brazil ^9^.

The CF-NBS emerged when Crossley et al. ^10^ reported that immunoreactive trypsinogen (IRT), the precursor biomarker of pancreatic enzymes, increased in the blood of most newborns with CF due to abnormal enzymatic drainage, regardless of whether the individual had pancreatic insufficiency ^10^ ^,^ ^11^. Currently, the initial stage of all NBS methods aims to determine IRT values. However, their second and third stages vary across geographic regions, depending on the incidence of the disease in the population, genetic background, intended targets, and the cost-effectiveness of the protocol, including saving resources by early diagnosis ^3^ ^,^ ^11^ ^,^ ^12^. It should be remembered that NBS is not only a screening test but also a public health program with several benefits for the population and large costs to the government. Thus, evidence for its efficacy must support such budget.

The Brazilian Unified Naional Health System (SUS, acronym in Portuguese) incorporated NBS in 2001 by creating the Brazilian National Neonatal Screening Program, which covers all 26 states and the Federal District of Brazil ^2^ ^,^ ^13^. One of the main objectives of the Brazilian National Neonatal Screening Program is to expand screening coverage to 100% of infants (or at least to 80%) ^14^. Its original plan had three steps: first, to screen for phenylketonuria and congenital hypothyroidism; second, for sickle cell anemia and other hemoglobinopathies; and third, for cystic fibrosis. In 2014, the Brazilian National Neonatal Screening Program included two new diseases: congenital adrenal hyperplasia and biotinidase deficiency. The Brazilian National Neonatal Screening Program has recently added congenital toxoplasmosis to its protocol, investigating a total of seven diseases ^15^.

Although the CF-NBS has been part of the Brazilian National Neonatal Screening Program since 2001, it is yet to be fully integrated in all states ^16^. The Brazilian Ministry of Health has adopted the IRT/IRT protocol with the collection of up to two IRT samples at different time points. Briefly, primary health care units collect the first IRT sample (IRT1). A second sample should be collected if the IRT1 value exceeds 70ng/mL. A sweat test (ST) must be performed if the level of IRT2 exceeds 70ng/mL or if the IRT2 is collected after the first 30 days of the newborn’s life. This screening IRT/IRT method is highly questionable because it depends on two samplings and has a low sensitivity and positive predictive value (PPV) ^17^ ^,^ ^18^.

If the PPV of a screening test or a combined screening test is considered too low, it could be adjusted by raising or lowering the cutoff values for a continuous variable or by changing the components that make up a screening strategy ^19^. Previous studies in the Brazilian Northeast and Southeast have shown that the IRT/IRT strategy has a low PPV and a high number of false positive cases ^20^ ^,^ ^21^. As a result, recalling individuals and performing additional clinical and laboratory tests dramatically increases the cost of the CF-NBS in a continental nation like Brazil.

At a time when the Brazilian Ministry of Health is discussing the expansion of the national newborn screening program by including new diseases in screening ^15^, it is important to improve the NBS strategies for the diseases that are already included in the Brazilian National Neonatal Screening Program and which still show room for improvement, such as CF.

Therefore, it is desirable that a study be conducted to determine the accuracy of the IRT/IRT protocol for CF-NBS in Brazil. Given the higher cost of using molecular techniques and the great genetic diversity ^22^ ^,^ ^23^ ^,^ ^24^ ^,^ ^25^, especially in Northeastern Brazil, this independent analytical study of the current cutoff points in the IRT/IRT strategy aimed to analyze a few alternative fixed and floating IRT1 and IRT2 cutoff points.

## Methods

### Design and study period

Descriptive, individual-level data were retrospectively collected from 2013 to 2017 for this observational population study.

### Population, site, and eligibility criteria

The Brazilian state of Bahia is located in Northeastern region. It has a population of approximately 15 million people. The studied years saw about 200,000 births per year. The state spans about 570,000km^2^ and contains 417 municipalities. All newborns that were cared for by SUS within the NBS program of the Brazilian National Neonatal Screening Program in Bahia were eligible for this study.

Neonates for whom no new IRT1 or IRT2 sample were obtained after inadequate IRT1 or IRT2 sampling were excluded. Individuals from whom one sample was collected after 30 days of their lives, were unable to complete the IRT/IRT protocol, or died without diagnostic confirmation were also excluded.

### Protocol for cystic fibrosis newborn screening in Bahia State (IRT/IRT)

The screening algorithm established in Bahia was in accordance with the recommendations of the Brazilian Group for Cystic Fibrosis Studies and the Brazilian Ministry of Health ^2^ ^,^ ^26^. IRT was performed at up to two different time points (IRT/IRT protocol) as part of the strategy. Dry blood spots were collected on filter paper in about 4,500 primary healthcare units. These samples were delivered to a reference laboratory for newborn screening in the state capital, Salvador. The recommended range to collect IRT1 varied from the 3rd to the 5th days of life ^2^. In cases of elevated IRT1 (≥ 70ng/mL), IRT2 was required and should ideally be collected from the 10th to the 21st days after birth. To collect a second sample of DBS from newborns with abnormal IRT1 levels, the NBS reference service contacted primary healthcare units. The sample was collected and delivered using the same procedure as the first IRT. If IRT2 was also elevated (≥ 70ng/mL), the infant should be referred to a CF care center for clinical evaluation and ST ^2^. Bahia has two CF care centers, both of which are in the capital. Sampling for IRT1 or IRT2 dosages in individuals older than 30 days must be considered inadequate and unreliable ^2^ ^,^ ^14^.

### Processing of samples on filter paper

Blood samples were dried on filter paper and processed on AutoDELFIA (Waltham, United States) using the PerkinElmer Neonatal IRT kit (PerkinElmer do Brasil, São Paulo, Brazil) and an automated immunofluorometric method.

### Sweat test

At the CF care centers, newborns were subjected to quantitative iontophoresis dosages using pilocarpine stimulation according to a predetermined algorithm. The sweat tests were interpreted as recommended ^26^ ^,^ ^27^. In total, two positive tests at different times in several samples were required to confirm the disease ^26^.

### Study variables and implementation

The variables of interest were the annual number of live births in the state and the number of confirmed cases of CF by ST. IRT1 and IRT2 levels and the number of CF infants with negative CF-NBS were also described.

Samples were considered late if the IRT1 was collected after 15 days of life and inadequate if the IRT1 or IRT2 was collected after 30 days of life ^2^ ^,^ ^26^. If the collected material was unable to be processed due to technical difficulties in collection, storage, or transport, the samples were also considered inadequate.

### Source and preparation of data

All data on the number of examined newborns, IRT values, and time to IRT1 and IRT2 results were provided from the NBS Reference Service computerized information system. The number of live births in the State of Bahia was retrieved for each study year from the website of the Information Technology Department of the SUS (DASTASUS, acronym in Portuguese) ^28^. To complete the database construction, the data were imported to Microsoft Excel for Mac, version 16.48 (https://products.office.com/).

### Data analysis

Stata, version 17.0 (https://www.stata.com); SPSS, version 21.0 (https://www.ibm.com/); Microsoft Excel for Mac, version 16.48; and OpenEpi (http://www.OpenEpi.com) ^29^ were used for data tabulation and analysis. Statistical descriptions of data using means and/or medians with related dispersions (amplitude, variance, standard deviation, variation coefficient, and confidence interval) for quantitative indicators and simple and relative frequency measures for categories were employed.

Newborns were classified as false positives if they had an elevated IRT1 sample (≥ 70ng/mL) and a negative ST (< 30mEq/L) or both a high IRT1 and IRT2 (≥ 70ng/mL) and a negative ST. False negatives were defined as infants with a negative CF-NBS (IRT1 < 70ng/mL) or elevated IRT1 but a IRT2 < 70ng/mL, clinical findings consistent with CF, and a disease diagnosis confirmed by ST.

Sensitivity, specificity, PPV, negative predictive value (NPV), and overall accuracy with 95% confidence intervals (95%CI) were analyzed. In the first step, to assess accuracy, the CF-NBS protocol IRT1/IRT2 was considered as a single test, with ST serving as the gold standard. In the second step, the nonparametric DeLong method was used to determine the receiver operator characteristic curve (ROC) for the annual assessment of IRT1 and IRT2 cutoff points. Using the binomial approach, the area under the curve (AUC) was calculated with the corresponding 95%CI. Sensitivity, specificity, and number of false positives were evaluated for each IRT1 and IRT2 cutoff point. The optimal cutoff point was defined based on the highest sensitivity and specificity according to the Youden’s index, which evaluates the ability of a diagnostic test to balance sensitivity (detection of disease) and specificity (detection of health or no disease). Therefore, the greatest Youden’s index value associated with a given IRT score was offered as the optimal cutoff value for each year, using all data from the previous year (e.g., the 2013 IRT Youden’s index was used as the 2014 IRT cutoff value). IRT1 and IRT2 values for the 99.4-, 99.5-, 99.6-, and 99.7-percentiles from the previous year were also evaluated to examine the influence of the sliding cutoff point. If the Youden’s index IRT values were close in all examined 5 years, a fixed point within the found variation was used for an isolated analysis. Because of this and since other studies recommended 90ng/mL ^18^ ^,^ ^30^ ^,^ ^31^, this value was used for IRT2.

### Ethical concerns

The data in this research were retrieved as part of another study ^21^ ^,^ ^32^ (CAAE: 79495717.1.0000.0049), which was subjected to the Research Ethics Committee of University Hospital Professor Edgard Santos and approved by it under number 2.389.852/2017. This study is in accordance with the research ethics principles of the Declaration of Helsinki and *Resolution n. 466/2012* of the Brazilian National Health Council.

## Results

**Figure 1:**
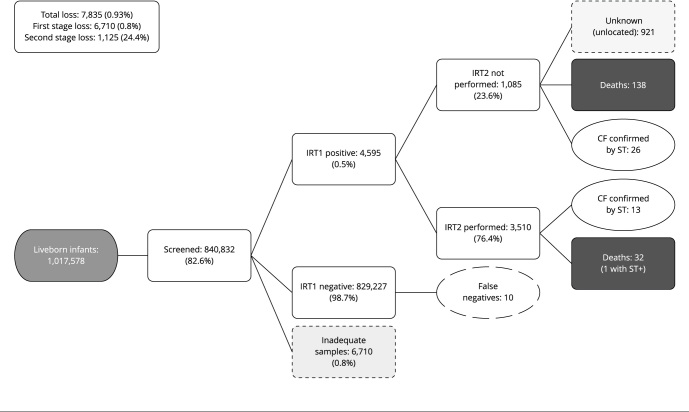
Results of screening for cystic fibrosis in newborns in Bahia State, Brazil (2013-2017).

CF: cystic fibrosis; IRT1/IRT2: first/second sample of immunoreactive trypsinogen; ST: sweat test. Source: based on Godoy et al. ^21^.

From 2013 to 2017, 1,017,576 live births occurred in Bahia, and 840,976 (82.6%) infants were subjected to CF-NBS. Of these, 6,640 (0.79%) IRT1 samples were classified as unsuitable for analysis. The second stage of CF-NBS included a 23.6% loss, totaling 3,510 infants subjected to IRT2 measurement (Figure 1).

During the study period, 49 infants were diagnosed with CF, 39 of whom were identified by NBS (79.6%). The remaining cases had CF-compatible symptoms and were subsequently confirmed as positive diagnoses, although screening results were initially negative (false negatives). A total of 13/39 (33.3%) children who tested positive for CF-NBS were correctly diagnosed in accordance with all steps of the diagnostic protocol (IRT1/IRT2/ST). Thus, the tests used to diagnose 26 infants consisted only of IRT1 and ST. Figure 1 shows an algorithm. A previous study has recently detailed these results ^21^.

**Figure 2:**
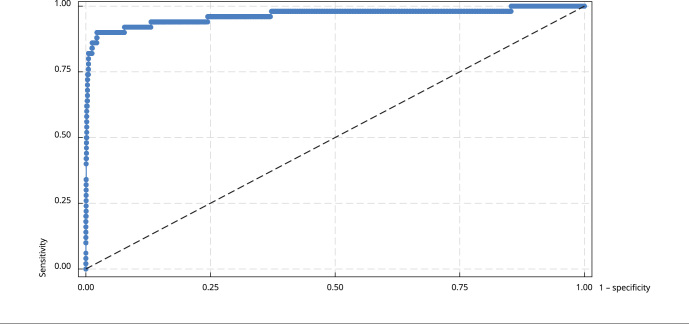
Receiver operating characteristic (ROC) curve and area under the curve (AUC) for the cumulative first sample of immunoreactive trypsinogen (IRT1).

The current CF-NBS (IRT1/IRT2) could predict the presence of disease in 99.93% (95%CI: 99.92-99.93) of cases, with a 79.59% sensitivity (95%CI: 66.36-88.52) and a 100% specificity (95%CI: 100-100) from 2013 and 2017. Table 1 shows the annual and five-year accuracy statistics for the CF-NBS (IRT/IRT) and shows the number of valid IRT1 and IRT2 after excluding inappropriate samples and cases of death. The number of valid IRT2 tests was less than the number of false-positive cases. The observed AUC for IRT1 totaled 0.96 (95%CI: 0.9635-0.9643). The ROC curve for IRT2 evaded construction due to the small number of tested newborns (n = 13 in 5 years). Figure 2 shows these data and the operator characteristic curve (ROC) for all years (2013 to 2017).

**Table 1:** Annual and cumulative accuracy assessment of the protocol to newborns screening for cystic fibrosis (IRT/IRT).

Variable	2013 (N)	2014 (N)	2015 (N)	2016 (N)	2017 (N)	All (N)
Subjected to CF-NBS	144,215	170,792	177,998	171,182	176,789	840,976
IRT1 valid	142,536	168,016	176,688	170,709	176,387	834,336
IRT 1 ≥ 70mg/dL	951	432	933	1,274	1,005	4,595
IRT 2 valid	655	356	637	1,004	858	3510
IRT 2 ≥ 70mg/dl	36	20	48	51	46	201
False positive	96	51	150	132	176	605
Total confirmed CF	8	7	12	11	11	49
Confirmed CF-NBS	7	5	9	11	7	39
Confirmed CF by clinical suspicion	1	2	3	0	4	10
	2013 [% (95%CI)]	2014 [% (95%CI)]	2015 [% (95%CI)]	2016 [% (95%CI)]	2017 [% (95%CI)]	All [% (95%CI)]
Accuracy	99.93 (99.92-99.94)	99.97 (99.96-99.98)	99.92 (99.9-99.93)	99.92 (99.91-99.93)	99.9 (99.88-99.91)	99.93 (99.92-99.93)
Sensitivity	87.5 (52.9-97.8)	71.43 (35.89-91.78)	75.0 (46.77-91.11)	100 (74.12-100)	63.64 (35.38-84.83)	79.59 (66.36-88.52)
Specificity	100 (100-100)	100 (100-100)	100 (100-100)	100 (100-100)	100 (99.9-100)	100 (100-100)
PPV	6.8 (3.3-13.4)	8.93 (3.87-19.26)	5.66 (3.01-10.41)	7.69 (4.35-13.25)	3.83 (1.87-7.68)	6.06 (4.46-8.17)
NPV	99.93 (99.92-99.94)	99.97 (99.96-99.98)	99.91 (99.9-99.93)	99.92 (99.91-99.93)	99.9 (99.88-99.91)	99.93 (99.92-99.93)

95%CI: 95% confidence interval; CF-NBS: newborn screening for cystic fibrosis; IRT1/IRT2: first/second sample of immunoreactive trypsinogen; PPV: positive predictive value; NPV: negative predictive value.

Area under the ROC curve = 0.9639.

For IRT1 performance, no proposed cutoff performed better than the current one (70ng/mL) (Table 2). The Youden’s index found that the best ratios between specificity and sensitivity were very close to 70.0ng/mL, as seen in three of the 5 years: 70.0ng/mL (2013), 70.4ng/mL (2015), and 71.3ng/mL (2016). This value was lower in the other 2 years: 36.5ng/mL (2014) and 59.7ng/mL (2017) (Table 2).

**Table 2:** Annual accuracy data for the first sample of immunoreactive trypsinogen (IRT1) at distinct cutoff points

Parameters	2013	2014 *	2015 **	2016 ***	2017 ^#^
Subjected to IRT1	N = 142,536	N = 168,016	N = 176,688	N = 170,495	N = 176,387
Identified by CF-NBS	7	5	9	11	7
Range IRT1 (CF-NBS confirmed) [minimum-maximum]	70.1-282.0	80.37-210.0	70.5-270.0	71.4-314.0	82.6-225.64
Identified by clinical suspicion (false negative)	1	2	3	0	4
Range of IRT1 (false negative) [minimum-maximum]	30.6	36.6-58.9	19.8-23.97	NA	10.25-59.68
Range of IRT1 (all) [minimum-maximum]	0.01-356.0	0.23-801.0	0.14-827.0	0.41-451.0	0.08-599.0
IRT1: 70ng/mL					
Sensitivity (%)	87.50 ^##^	55.60	83.33	100.0	80.0
Specificity (%)	99.35 ^##^	99.75	99.47	99.26	99.43
False positive (%)	0.65 ^##^	0.25	0.53	0.74	0.57
False positive (n)	923 ^##^	417	940	1,268	1,004
IRT1 (ng/mL): 99.4^th^ percentile	71.60	60.10	66.29	74.4	68.35
Sensitivity (%)	NA	55.56	83.33	100.0	80.0
Specificity (%)	NA	99.77	99.09	99.08	99.57
False positive (%)	NA	0.23	0.91	0.92	0.43
False positive (n)	NA	393	1,599	1,562	760
IRT1 (ng/mL): p99.5^th^ percentile	75.9	61.90	71.90	79.60	72.10
Sensitivity (%)	NA	55.56	83.33	90.91	80.0
Specificity (%)	NA	99.81	99.21	99.33	99.67
False positive (%)	NA	0.19	0.79	0.67	0.33
False positive (n)	NA	324	1,391	1,140	587
IRT1 (ng/mL): p99.6^th^ percentile	80.9	63.60	77.8	85.6	76.20
Sensitivity (%)	NA	55.56	83.33	81.82	70.0
Specificity (%)	NA	99.84	99.30	99.47	99.74
False positive (%)	NA	0.16	0.70	0.53	0.26
False positive (n)	NA	277	1,235	906	454
IRT1 (ng/mL): p99.7^th^ percentile	86.34	66.80	85.79	93.80	82.28
Sensitivity (%)	NA	55.56	83.33	72.73	70.0
Specificity (%)	NA	99.86	99.42	99.61	99.81
False positive (%)	NA	0.14	0.58	0.39	0.19
False positive (n)	NA	228	1,017	665	327
IRT1 (ng/mL): Youden index	70.0	36.54	70.4	71.3	59.67
Sensitivity (%)	NA	55.6	83.33	100.0	80.0
Specificity (%)	NA	99.75	94.84	99.26	99.47
False positive (%)	NA	0.25	5.16	0.74	0.53
False positive (n)	NA	417	9,118	1,254	935

CF-NBS: newborn screening for cystic fibrosis; NA: not applicable.

* The 2013 IRT1 value was used as the percentile;

** The 2014 IRT1 value was used as the percentile;

*** The 2015 IRT1 value was used as the percentile;

^#^ The 2016 IRT1 value was used as the percentile;

^##^ Calculated accuracy using the 2013 IRT1 value

IRT2 performance with floating cutoffs seems to be unapplicable. The use of the Youden’s index indicated that the IRT2 cutoff point appears to be more effective at higher cutoff points, possibly reducing the proportion of false-positive results (Table 3). However, it was impossible to obtain these data for each year as only a few children underwent all CF-NBS steps (IRT1/IRT2). Nonetheless, when these measurements were available, the Youden’s index found the ideal cutoff values to total 94.8ng/mL (2013), 116.0ng/mL (2014), 97.6ng/mL (2015), and 104ng/mL (2017). Thus, a fixed 90ng/mL cutoff served to analyze the data, showing the same sensitivity and specificity as the current cutoff (70ng/mL) but with fewer false positives (Table 3).

**Table 3:** Annual accuracy data for the second sample of immunoreactive trypsinogen (IRT2) at distinct cutoff points.

Parameters	2013	2014 *	2015 **	2016 ***	2017 ^#^
Subjected to IRT2	N = 655	N = 356	N = 637	N = 1,004	N = 858
Identified by CF-NBS	3	3	4	0	3
Range IRT2 (all) [minimum-maximum]	6.19-410.5	0.19-492.0	1.59-385.0	1.92-671.0	1.15-273.0
IRT2: 70ng/mL					
Sensitivity (%)	100.0 ^##^	100.0	100.0	NA	100.0
Specificity (%)	94.94 ^##^	95.18	93.04	NA	94.86
False positive (%)	5.6 ^##^	4.82	6.96	NA	5.14
False positive (n)	33 ^##^	17	44	NA	44
IRT2: 90ng/mL					
Sensitivity (%)	100.0 ^##^	100.0	100.0	NA	100.0
Specificity (%)	97.24 ^##^	97.17	97.31	NA	96.85
False positive (%)	2.76 ^##^	2.83	2.69	NA	3.15
False positive (n)	18 ^##^	10	17	NA	27
IRT2 (ng/mL): 99.4^th^ percentile	163.57	189.58	296.42	232.19	197.15
Sensitivity (%)	NA	33.3	75.0	NA	0
Specificity (%)	NA	98.87	99.37	NA	99.53
False positive (%)	NA	1.13	0.63	NA	0.47
False positive (n)	NA	4	4	NA	4
IRT2 (ng/mL): 99.5^th^ percentile	169.48	255.71	301.52	282.25	235.02
Sensitivity (%)	NA	0	50.0	NA	0
Specificity (%)	NA	98.87	99.53	NA	99.65
False positive (%)	NA	1.13	0.47	NA	0.35
False positive (n)	NA	4	3	NA	3
IRT2 (ng/mL): 99.6^th^ percentile	172.76	363.17	333.74	290.90	278.33
Sensitivity (%)	NA	0	25.0	NA	0
Specificity (%)	NA	99.15	99.84	NA	99.65
False positive (%)	NA	0.85	0.16	NA	0.35
False positive (n)	NA	3	1	NA	3
IRT2 (ng/mL): 99.7^th^ percentile	181.57	470.62	372.25	310.29	302.38
Sensitivity (%)	NA	0	0	NA	0
Specificity (%)	NA	99.43	100.0	NA	99.88
False positive (%)	NA	0.57	0	NA	0.12
False positive (n)	NA	2	0	NA	1
IRT2 (ng/mL): Youden index	94.8	116	97.6	NA	104
Sensitivity (%)	NA	100.0	75.0	NA	100.0 ^###^
Specificity (%)	NA	97.17	98.42	NA	97.31 ^###^
False positive (%)	NA	2.83	1.58	NA	2.69 ^###^
False positive (n)	NA	10	10	NA	23 ^###^

CF-NBS: newborn screening for cystic fibrosis; NA: not applicable.

* The 2013 IRT2 value was used as the percentile;

** The 2014 IRT2 value was used as the percentile;

*** The 2015 IRT2 value was used as the percentile;

^#^ The 2016 IRT2 value was used as the percentile;

^##^ Calculated accuracy using the 2017 IRT2 value;

^###^ Calculated accuracy using the 2015 IRT2 value.

## Discussion

This study analyzed data from 840,976 infants subjected to CF-NBS from 2013 to 2017 in the State of Bahia. Of the 49 CF diagnosis, 39 were due to positive screenings and 10, to clinical suspicion − showing a negative CF-NBS (false negative). The accuracy of the screening protocol (IRT/IRT) for this specific population, with a 70ng/mL cutoff point, totaled 99.93%, with a 79.6% sensitivity, a 99.9% specificity, and a 6.1% PPV. When studying changes in cutoff values and the use of floating cutoff points of IRT2, this study found that a cutoff point near 90mg/mL seemed to provide the same sensitivity and specificity but with an improved PPV, resulting in a 46.7% (±10.2) decrease in the percent mean (standard deviation − SD) of false-positive results, with a 38.3% (2017) and 61.4% (2015) minimum and maximum reduction, respectively.

This study found a variation in sensitivity over time. Values were consistently lower than the minimum 95% recommended by the European Cystic Fibrosis Society (ECFS) ^33^. This level of sensitivity suggests that the screening protocol used is scarcely successful in identifying individuals with the disease, thereby increasing the number of false-negative results. This study highlights the challenge of using this protocol to identify CF in newborns due to the high percentage of false-negative results (20.4%). Furthermore, this strategy showed a greater proportion of false-positive outcomes, which is associated with higher costs and a psychological burden for governments and families, respectively ^18^ ^,^ ^34^ ^,^ ^35^. This is particularly relevant in states such as Bahia, which has considerable distances between most municipalities and the NBS service or CF care centers. Many other Brazilian states show the same reality.

Other studies have shown that the CF-NBS IRT/IRT protocol had a false-negative rate from 5 to 15% ^20^ ^,^ ^36^. The genetic heterogeneity of the studied population may be a contributing factor to the higher number of false-negative results, which makes it difficult to determine the optimal cutoff points for this community ^23^ ^,^ ^25^ ^,^ ^37^ ^,^ ^38^. A study conducted in Argentina evaluated the IRT/IRT protocol and found 14% of false-negative results with a 80% sensitivity. However, its sample size was much smaller than that in this study ^31^. Studies conducted in Brazil, such as Maciel et al. ^20^, also found a low 1.2% VPP with a false negative rate of 11.5%. Similar data have been found in Andalusia ^17^ and Argentina ^31^. However, in all cases, the incidence of the disease exceeded that of the studied population, with rates of 1:6,675; 1:4,893 and 1:8,170 live births, respectively. Thus, the results of this study show good sensitivity and specificity (comparable to the other studies mentioned above) and a low PPV due to the lower incidence of the disease in our population. This highlights the importance of the methodological difficulties inherent in the test and the influence of epidemiologic conditions on the results of this study. The occurrence of false-negative outcome delays the CF diagnosis and may contribute to its worse prognosis ^39^.

The low PPV values in this study are comparable to those in previous studies and seem to be related to the used methods ^30^ ^,^ ^40^. ECFS guidelines recommend that the minimum acceptable PPV for CF-NBS should totaled 30% (0.3), significantly higher than the value in this study ^33^. Nevertheless, it is important to note that the validity of these recommendations may be limited in communities with a significantly low incidence of CF. This is particularly relevant for the studied population, for which the incidence of the disease is estimated to total about 1:20,000 live births ^21^. Therefore, despite the use of protocols with proven efficacy and high specificity, PPV tends to be consistently lower than the value the ECFS reported as it is also directly related to the incidence/prevalence of the disease ^19^.

Indeed, the high number of infants referred to ST despite being freed from the disease contributed to the increased costs of screening at SUS. Moreover, this situation placed a significant psychosocial burden on families until a definitive diagnosis was confirmed or ruled out ^34^. This is primarily due to the low PPV associated with the screening process ^7^ ^,^ ^35^ ^,^ ^41^. Even if the recommended PPV parameters are unsatisfied, decreasing the number of false-positive tests is a goal of the CF-NBS improvement initiative.

This study evaluated alternative cutoff points for IRT1 and the possibility of using floating cutoff points. However, the suggest that these options failed to be more effective than the current cutoff point. The low incidence of CF in the studied population may have acted as a negative determinant that made using floating cutoff points more difficult. When considering the decision to use brief time intervals for implementing the floating cut point approach, it is highly likely that CF was detected in a few infants, if any at all, making the calculation almost unattainable.

Although several studies suggest that the floating cutoff points increase testing efficiency and reduce the difficulties associated with IRT changes due to the heterogeneous genetic variants in populations, this study found otherwise ^38^ ^,^ ^42^ ^,^ ^43^. IRT values significantly change with temperature, evident information in temperate regions for which the floating cutoff points seem more desirable ^44^ ^,^ ^45^. The ineffectiveness of floating cutoff points may be attributable to the fact that this investigation was conducted in a tropical zone location with little change in annual temperature.

Based on the data obtained using the Youden’s index as a method to choose the ideal IRT1 cutoff point for each year, it seemed that this value was significantly lower than the current fixed cutoff point in 2014 and 2017. It is worth considering whether these data indicate a realistic potential for lowering the IRT1 cutoff point to increase the sensitivity of the test by attempting to reduce the number of false-negative results. However, the performed analyses showed no evidence of improvement to the test sensitivity and specificity, suggesting that the change would unlikely increase the cost-effectiveness of CF-NBS. The best ratios between specificity and sensitivity for IRT1 in other years being very close to 70.0ng/mL confirm this result.

Lowering the IRT1 cutoff value effectively increases sensitivity and decreases the number of false-negative results ^19^. On the other hand, the number of false positives will rise, worsening the CF-NBS program and adding significantly to SUS and family expenditures ^46^ ^,^ ^47^. In fact, municipalities such as Colorado and Buenos Aires, which adopted the same IRT/IRT methodology as this study, have implemented 50ng/mL IRT1 cutoff values, experiencing the anticipated effects ^31^ ^,^ ^43^. he first stage of the procedures in a Belgian investigation used a similar 60ng/mL fixed IRT limit, but the protocol of that study employed molecular techniques ^48^. Our data and analysis indicate that this approach seems inappropriate. If this change is considered, it is important to act prudently and weigh the risks and advantages of such alteration.

As previously mentioned, using the Youden’s index to determine the ideal IRT2 cutoff point showed that, in three of the five assessed years, this number was higher than the one currently in use. Raising the IRT2 cutoff to 90ng/mL (fixed) preserves the sensitivity and specificity of the test and significantly decreases the incidence of false positives. These findings may suggest that the protocol of CF-NBS could be modified by increasing the IRT2 thresholds to 90 to 100ng/mL, which would likely have a positive impact. However, this datum must be viewed with great caution considering the small number of infants who underwent IRT2 measurement ^18^ ^,^ ^49^ ^,^ ^50^.

There are other strategies to improve the accuracy of CF-NBS. Although the use of molecular methods for CF-NBS helps to increase the sensitivity of the test, it is important to recognize the limitations of its use in countries with limited resources, low incidence of the disease, and especially a high rate of CFTR gene heterogeneity. In 2003, Raskin et al. ^24^ showed high allele variability among Brazilian children with CF, making it difficult to import variant panels from other countries. In Bahia, a study showed that a panel including 20 variants was required to cover 90% of the CF infants in this population, detecting a previously undescribed mutation ^38^. Moreover, allele variation among Brazilian states is large, so each locality would have to create its own CF gene panel ^51^. Although the costs of molecular biology technologies have recently decreased, they remain steep, affecting the cost-effectiveness of this public health intervention and hindering its implementation in Brazilian states with limited government resources and lower human development indices. Thus, new strategies must be considered in this scenario.

Beyond examining variants in the CFTR gene, other screening strategies, such as pancreatitis-associated protein (PAP), should be considered as alternatives to alleviate the problems of the IRT/IRT strategy. The use of PAP associated with IRT without molecular technologies has shown an excellent cost-benefit ratio because it reuses the same dry blood stop sample that was collected during the initial test ^31^ ^,^ ^46^ ^,^ ^48^ ^,^ ^52^, eliminating the need for a another blood draw in the primary care unit ^52^ ^,^ ^53^. Several European countries have included the PAP approach with positive results in their screening protocols ^30^ ^,^ ^48^ ^,^ ^54^ ^,^ ^55^. In Argentina, a comparison between IRT/IRT and IRT/PAP showed improved sensitivity and reduced number of false negatives ^31^. However, that study was conducted with a small sample limited to the Buenos Aires population. Brazil requires further studies to determine whether the use of PAP is feasible.

It is important to mention the use of retrospective secondary data and screening-related losses as limitations of this study. Moreover, IRT2 analysis results should be considered with caution due to the small number of infants who completed all CF-NBS phases, with the IRT1/IRT2/ST procedures performed on a tiny minority of newborns. Because IRT2 sample collection occurred after 30 days of life in a large proportion of infants, only 53% of CF newborns underwent IRT1/ST. This explains the lower number of valid IRT2 tests than that of false positive tests. It should also be emphasized that the data refer only to the first 5 years of the NBS in Bahia and may not have been fully adequate for a proper evaluation of the accuracy of a complex and important public health program such as newborn screening.

In conclusion, this study indicates that the current CF-NBS protocol applied to the Bahia population has both low sensitivity and predictive positive value, with a significant number of false positives and negatives. The introduction of a floating cutoff point for IRT1 or IRT2 seems to offer an unviable option for the studied population. Changing the IRT2 cutoff point from 70ng/mL to values between 90ng/mL and 100ng/mL must have advantages and should be considered with caution. This measure fails to alter this routine and has no overall cost to SUS. A reduction in the IRT1 threshold has been proposed. However, the results of this study preclude the precise definition of a value. Any such adjustment should be carefully considered as it may do more harm than good. Implementation of new strategies with PAP or CFTR DNA analysis requires a pilot study to confirm this hypothesis.

Although far from being ideal, the current strategy is successful and should be maintained as a CF-NBS since promoting and encouraging NBS evaluation programs are crucial steps to continuously improve public policies, which must be done often. Further studies must be conducted to evaluate screening methods to selecting the method with the best cost-benefit ratio considering local disease incidence and the genetic and phenotypic characteristics of its population.
